# Effects of Sodium Lactate Infusion in Two Girls with Glucose Transporter 1 Deficiency Syndrome

**DOI:** 10.1055/a-2134-8766

**Published:** 2023-09-22

**Authors:** Loes A. van Gemert, Nens van Alfen, Lizzy van Gaal, Saskia Wortmann, Michèl A. Willemsen

**Affiliations:** 1 Department of Pediatric Neurology, Amalia Children's Hospital, Donders Institute for Brain, Cognition and Behaviour, Radboud University Medical Center, Nijmegen, The Netherlands; 2 Department of Neurology and Clinical Neurophysiology, Donders Institute for Brain, Cognition and Behaviour, Radboud University Medical Center, Nijmegen, The Netherlands; 3 University Childrens Hospital, Paracelsus Medical University Salzburg, Salzburg, Austria; 4 Department of Metabolic Diseases, Radboud University Medical Center, Nijmegen, The Netherlands

**Keywords:** glucose transporter 1 deficiency syndrome, sodium lactate infusion, epilepsy, electroencephalography, experimental therapy

## Abstract

**Background** Glucose is an important fuel for the brain. In glucose transporter 1 deficiency syndrome (GLUT1DS), the transport of glucose across the blood–brain barrier is limited. Most individuals with GLUT1DS present with developmental problems, epilepsy, and (paroxysmal) movement disorders, and respond favorably to the ketogenic diet. Similar to ketones, lactate is an alternative energy source for the brain. The aim of this study is to investigate whether intravenous infusion of sodium lactate in children with GLUT1DS has beneficial effects on their epilepsy.

**Methods** We performed a proof of principle study with two subjects with GLUT1DS who were not on a ketogenic diet and suffered from absence epilepsy. After overnight fasting, sodium lactate (600 mmol/L) was infused during 120 minutes, under video electroencephalographic (EEG) recording and monitoring of serum lactate, glucose, electrolytes, and pH. Furthermore, the EEGs were compared with pre-/postprandial EEGs of both subjects, obtained shortly before the study.

**Results** Fasting EEGs of both subjects showed frequent bilateral, frontocentral polyspike and wave complexes. In one subject, no more epileptic discharges were seen postprandially and after the start of lactate infusion. The EEG of the other subject did not change, neither postprandially nor after lactate infusion. Serum pH, lactate, and sodium changed temporarily during the study.

**Conclusion** This study suggests that sodium lactate infusion is possible in individuals with GLUT1DS, and may have potential therapeutic effects. Cellular abnormalities, beyond neuronal energy failure, may contribute to the underlying disease mechanisms of GLUT1DS, explaining why not all individuals respond to the supplementation of alternative energy sources.

## Introduction

Glucose, the main source of energy for the brain, is transported across the blood–brain barrier by glucose transporter protein type 1 (GLUT1). 1 2 3 In GLUT1 deficiency syndrome (GLUT1DS), glucose uptake into the brain is limited due to decreased glucose transport capacity, leaving the brain with a permanent energy deficit. GLUT1DS is caused by heterozygous, often *de novo* , pathogenic variants in the *SLC2A1* gene. Low cerebrospinal fluid glucose concentrations in the context of normoglycemia, with normal to low cerebrospinal fluid (CSF) lactate concentrations, reflect the underlying disease mechanism of GLUT1DS, and are pivotal laboratory findings in establishing the diagnosis. 4 5 6

Drug-resistant epilepsy, paroxysmal movement disorders, and developmental delay are symptoms of the classic phenotype of GLUT1DS; however, individuals with mono- or oligosymptomatic phenotypes are increasingly recognized. 5 7 8 Typically, symptoms are worst after a fasting period, for example, in the morning before breakfast. Paroxysmal exercise-induced dyskinesia (PED) generally occurs after voluntary movement. The majority of patients respond well to ketogenic diet therapy (KDT), which provides the brain with ketone bodies as an alternative source of energy. Nevertheless, not all patients respond well to KDT. 9 10

Lactate can provide up to 8 to 10% of the brain's energy requirement under certain clinical circumstances, 11 and even more when serum lactate is artificially raised. 12 13 14 15 The underlying cellular mechanism can be explained by the presence of the so-called astrocyte neuron lactate shuttle, stating that astrocytes convert glucose into lactate through aerobic glycolysis, and subsequently shuttle lactate as an energy source toward neurons. 16 17

Importantly, in clinical practice, lactate is generally seen as a waste product and marker of severe, critical illness; there is little attention for its potential therapeutic application. Nevertheless, the therapeutical use of sodium lactate infusion has been investigated in a few clinical studies. 18 We hypothesized that lactate could serve as an energy source for the brain in individuals with GLUT1DS and that its application would lead to an almost immediate decrease of quickly reversible symptoms and signs, like epileptic seizures and epileptiform discharges on electroencephalogram (EEG). To test this hypothesis, we conducted a proof of principle study with sodium lactate infusion in two children with GLUT1DS, monitored with video EEG.

## Methods

We performed a single-center, interventional, explorative, open label, proof of principle study. The study was approved by the institutional review board of the Radboud University Medical Center and the Central and Regional Committee on Research Involving Human Subjects (reference 2020-6231) and was performed in accordance with the tenets of the Declaration of Helsinki. Participating subjects and parents/caregivers gave written informed consent.

### Inclusion and Exclusion Criteria

Patients were eligible for this study if they had a diagnosis of GLUT1DS confirmed by a CSF profile that met the criteria for GlUT1DS and/or identification of a pathogenetic heterozygous *SLC2A1* variant. 19 Given the nature of this study, subjects with GLUT1DS who did not tolerate or did not respond to KDT were considered eligible. 9 They had to be at least 12 years old, and had to suffer from frequent, abundant or continuous seizure activity according to the American Clinical Neurophysiology Society (ACNS) guidelines. 20 The exclusion criteria were the following: following KDT during the last year prior to this study, any additional chronic or acute (at the moment of the study) medical condition, an increased serum sodium concentration (above 145 mmol/L), and a history of an anxiety disorder.

### Study Protocol

The subjects came to the hospital twice, both times after an overnight fast. During the first visit, they underwent video EEG recording using a standard 10- to 20-electrode montage. 21 Every 15 minutes, a 30-second artifact-free epoch in the awake state with eyes closed was selected from the EEG recording for formal assessment by an expert neurophysiologist (NvA) of the background activity, and for the presence of epileptiform discharges with or without a clinical correlate. In addition, the full EEG was evaluated for other changes that might have been missed by sampling the 30-second epochs. During epileptiform discharges greater than 5 seconds, the subjects were tested clinically by a word recall test. Furthermore, a hyperventilation test was performed twice to provoke epileptiform discharges. After 25 minutes, the subjects consumed a very carbohydrate-rich meal (within 15–30 minutes). After the meal, video EEG recording continued for another 25 minutes, in which the hyperventilation tests were repeated. This segment was evaluated in the same manner as the premeal EEG.

During the second visit, the subjects again underwent a video EEG recording using the standard 10- to 20-electrode montage. 21 Recordings started after a venous cannula was inserted, around 10 a.m. To obtain baseline recordings, video EEG recordings were started. These recordings were continued during and after infusion of sodium lactate until the subject's serum lactate concentration was normalized. The subjects remained in the fasting state until 12.30 p.m. The video EEG recording continued after the end of the sodium lactate infusion for at least 60 minutes, and until serum lactate concentrations were normalized, and then evaluated offline as outlined earlier. The subjects left the hospital after an additional observation period of at least 60 minutes, without any special treatment.

### Sodium Lactate Infusion and Biochemical Monitoring

The subjects received infusion of 600 mmol/L sodium lactate (Apotheek A15, Gorinchem, the Netherlands, prepared by the Department of Pharmacy, Radboud University Medical Center, Nijmegen, the Netherlands). We aimed for a lactate blood concentration of approximately 6 to 10 mmol/L and, based on the infusion protocols used in previous studies, 18 first primed subjects with an infusion rate of 0.10 mmol/kg bodyweight/min for 15 minutes, and continued with an infusion rate of 0.06 mmol/kg bodyweight/min for another 105 minutes. Before, during, and after infusion of sodium lactate, blood samples were collected to monitor changes in pH, base excess, sodium, potassium, chloride, bicarbonate, lactate, and glucose. At baseline, the first blood sample was drawn via the venous cannula, before sodium lactate infusion started. The rest of the blood testing was conducted by capillary sampling using finger pricks.

## Results



**Video 1** EEGs of subjects 1 and 2.


Five patients known in our center were eligible for this study. We included two 12-year-old girls diagnosed with GLUT1DS and both suffering from absence epilepsy (see Table 1 and Bekker et al 9 for additional clinical data). Both subjects had been on KDT in the past, but had stopped more than a year prior to the start of this study. Subject 1 had never reached full ketosis or symptom relief, but stopped the diet prematurely because of gastrointestinal side effects. Subject 2 had never benefitted from KDT despite adequate ketosis. At the moment of the present study, subject 1 used 250 mg acetazolamide twice a day, and subject 2 used lamotrigine 100 mg twice a day as antiseizure medication.

**Table 1:** Subject characteristics

Subject number	Sex	Weight (kg)	Genetic variant in *SLC2A1* (NM_006516)	Glucose serum (mmol/L)	Glucose CSF (mmol/L)	Lactate CSF (mmol/L)	CSF/blood glucose ratio	E	MD	FSIQ (PIQ, VIQ)	Subject number in Bekker et al 9
1	F	46	c.457C > T (p.(Arg153Cys))	5.4	2.0	1.090	0.37	+	PED, ataxia	82 (78/88)	2
2	F	56	Unknown b	4.9/3.4	2.4/2.3 a	1.6/1.057	0.49/0.68	+	–	88 (100/83)	7

Abbreviations: CSF, cerebrospinal fluid; E, epilepsy; FSIQ, full scale intelligence score; MD, movement disorder; PED, paroxysmal exercise-induced dyskinesia; PIQ, performance intelligence score; VIQ, verbal intelligence score.

a CSF analysis was done while on ketogenic diet therapy (KDT); the subject had asymptomatic hypoglycemia while on KDT.

b In the absence of an SLC2A1 gene mutation, CSF analysis was repeated to exclude transient cerebral glucose deficiency.

The baseline (fasting) EEGs were similar for subjects 1 and 2, showing frequent bilateral, frontocentral polyspike and wave complexes, with high amplitudes and durations between 2 and 10 seconds. Clinically, both girls had absence seizures during this part of the registration.

During the first visit, the postprandial EEG recording of subject 1 showed almost no epileptiform discharges anymore, and clinically, no more seizures were seen. The postprandial EEG at the first visit of subject 2, however, remained unchanged compared to the baseline EEG, and she also had seizures after breakfast. Hyperventilation did not provoke epileptiform discharges before or after the carbohydrate-rich meal in either subject.

**Fig. 1:**
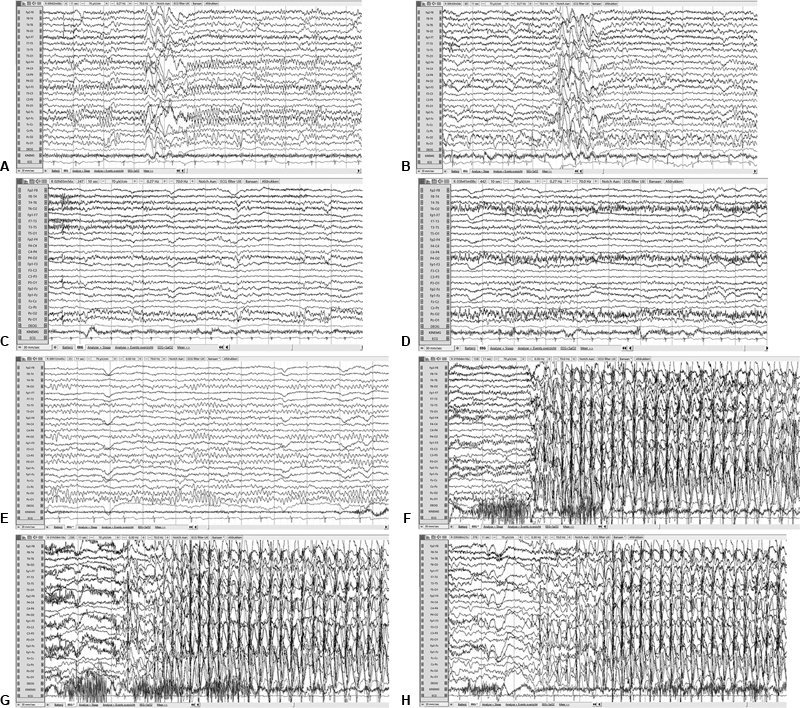
Fragments of the electroencephalogram (EEG) of subjects 1 and 2 during the second hospital visit: before, during, and after sodium lactate infusion. Subjects ( **A–D** ) 1 and ( **E–H** ) 2 before the start of sodium lactate infusion ( **A** and **E** ), while hyperventilating during sodium lactate infusion of 0.10 mmol/kg/min ( **B** and **F** ), while hyperventilating during sodium lactate infusion of 0.06 mmol/kg/min ( **C** and **G** ), and while hyperventilating after sodium lactate infusion has stopped ( **D** and **H** ).

In Fig. 1 , parts of the EEG recordings of the second visit of subjects 1 and 2, before and after sodium lactate infusion, are shown. Total amounts of approximately 360 and 440 mmol sodium lactate were infused in subjects 1 and 2, respectively. A video of the patients' EEGs of both visits are shown in Video 1 . The background activity of subject 1 was normal for her age. Occasionally, the EEG recording showed frontal epileptic discharges with spike-and-wave complexes and isolated spike and waves. No epileptiform discharges were seen in the EEG anymore from 40 minutes after the start of lactate infusion. The epileptic discharges and clinically recognizable absences did not reoccur on the day of the study. The background activity of subject 2 was also normal for her age. During her EEG recording, multiple absence seizures were clinically observed, with corresponding epileptiform discharges in the EEG. There was no change in seizures, neither clinically nor on the EEG recording of subject 2, during or after lactate infusion.

**Fig. 2:**
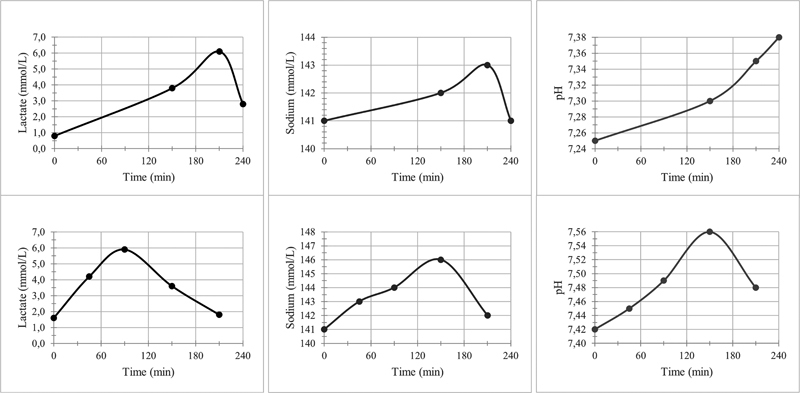
Serum lactate and sodium concentrations, and serum pH, of both subjects during the study with lactate infusion. In both subjects, sodium lactate was infused from *t* = 0 to 120 minutes.

Serum pH, lactate, and sodium concentrations did temporary increase, as expected, during the study period in both subjects ( Fig. 2 ). Subject 1 reported a tingling sensation in both arms, legs, and her face, developing a few minutes immediately after stopping the lactate infusion. The tingling sensation spontaneously disappeared after 2.5 hours.

## Discussion

This study suggests that sodium lactate infusion is possible in individuals with GLUT1DS, and may have a potential therapeutic effect. In subject 1, the clinical seizures and the epileptic discharges on EEG completely disappeared after lactate infusion, while no changes were observed in subject 2. Our observations in subject 1 suggest that lactate may indeed serve as an alternative fuel for the brain in GLUT1DS, like ketone bodies do.

As there are no previous studies to compare our results with, we do not know the potential response variability after “energy suppletion” in individuals with GLUT1DSl. There are, however, several studies that substantiated an improvement in epilepsy after breakfast, which also showed an improvement of EEG features after a meal or glucose infusion. 22 23 24 25 26 27 28 29 These studies included in total 26 children in the age range of 4 to 19 years. An improvement in the EEG was seen in 20/26 children. EEG improvements occurred after 4 minutes to 2 hours postprandially or after infusion of glucose. Our observations in subject 1 are in line with these data, confirming that in her lactate may improve brain function just as glucose does, at least for a relatively short period of time.

Interestingly, the EEG of subject 2 did not improve, neither after a meal nor after lactate infusion; she had also not benefitted previously from KDT. The findings in subject 2 may indicate that her brain suffers from a problem that cannot be solved by offering carbohydrates (breakfast) or alternative energy sources (lactate or ketone bodies), and as such confirm that additional cellular abnormalities, on top of the chronic energy failure, may contribute to the underlying disease mechanisms of GLUT1DS. 30 31 32 33

There are several limitations to this study. Since this was a proof of principle study, ethical guidelines allowed us to include only two patients. This precluded us from drawing conclusions on the effectiveness and safety of sodium lactate infusion as a treatment for the epilepsy of individuals with GLUT1DS in general. Additionally, these ethical guidelines did not allow the use of magnetic resonance spectroscopy (MRS) or positron emission tomography (PET) scans at this stage of clinical research, so we were unable to provide direct proof that the lactate was taken up by the brain. Next, assuming that lactate would be an alternative energy source for the brain, intravenous lactate infusion would not be a practical chronic treatment and may cause undesired side effects in the long term. However, this study presents the beneficial side of lactate and sheds light on the complex underlying disease mechanisms of GLUT1DS. By doing so, it gives a potential new direction in developing therapeutic possibilities in GLUT1DS. Interestingly, a similar way of reasoning motivated other researchers to investigate the effects of erythrocyte exchange transfusion in three patients with GLUT1DS. 34 Finally, pediatric patients with other (neurometabolic) disorders may possibly benefit from the observation that lactate can be used therapeutically in some conditions.

In conclusion, this study might suggest that lactate infusion has the potential to offer a safe alternative source of energy for the brain in individuals with GLUT1DS, without major side effects, at least when used for a short period. However, further research is needed to confirm these observations. Next, it is important to learn how to use this knowledge while exploring potential new therapeutic possibilities for individuals with GLUT1DS.
